# Human Menstrual Blood‐Derived Stem Cells Ameliorate Liver Fibrosis in Mice by Targeting Hepatic Stellate Cells via Paracrine Mediators

**DOI:** 10.5966/sctm.2015-0265

**Published:** 2016-07-28

**Authors:** Lijun Chen, Chunfeng Zhang, Lu Chen, Xiaojun Wang, Bingyu Xiang, Xiaoxing Wu, Yang Guo, Xiaozhou Mou, Li Yuan, Bo Chen, Jinfu Wang, Charlie Xiang

**Affiliations:** ^1^ Institute of Cell and Development, College of Life Sciences, Zhejiang University, Hangzhou, People's Republic of China; ^2^ State Key Laboratory for Diagnosis and Treatment of Infectious Diseases and Collaborative Innovation Center for Diagnosis and Treatment of Infectious Diseases, the First Affiliated Hospital, School of Medicine, Zhejiang University, Hangzhou, People's Republic of China; ^3^ Molecular Diagnosis Division, Zhejiang‐California International Nanosystem Institute, Zhejiang University, Hangzhou, People's Republic of China; ^4^ Clinical Research Institute, Zhejiang Provincial People's Hospital, Hangzhou, People's Republic of China; ^5^ Institute for Cell‐Based Drug Development of Zhejiang Province, Hangzhou, People's Republic of China

**Keywords:** Menstrual blood‐derived stem cells, Cellular therapy, Liver fibrosis, Stem cell transplantation, Paracrine protein factors

## Abstract

Mesenchymal stem cells (MSCs) may have potential applications in regenerative medicine for the treatment of chronic liver diseases (CLDs). Human menstrual blood is a novel source of MSCs, termed menstrual blood‐derived stem cells (MenSCs). Compared with bone marrow MSCs, MenSCs exhibit a higher proliferation rate and they can be obtained through a simple, safe, painless procedure without ethical concerns. Although the therapeutic efficacy of MenSCs has been explored in some diseases, their effects on liver fibrosis are still unclear. In the present study, we investigated the therapeutic effects of MenSC transplantation in a carbon tetrachloride‐induced mouse model of liver fibrosis. These results revealed that MenSCs markedly improved liver function, attenuated collagen deposition, and inhibited activated hepatic stellate cells up to 2 weeks after transplantation. Moreover, tracking of green fluorescent protein‐expressing MenSCs demonstrated that transplanted cells migrated to the sites of injury, but few differentiated into functional hepatocyte‐like cells. Transwell coculturing experiments also showed that MenSCs suppressed proliferation of LX‐2 cells (an immortalized hepatic stellate cell line) through secretion of monocyte chemoattractant protein‐1, interleukin‐6, hepatocyte growth factor, growth‐related oncogene, interleukin‐8, and osteoprotegerin. Collectively, our results provided preliminary evidence for the antifibrotic capacity of MenSCs in liver fibrosis and suggested that these cells may be an alternative therapeutic approach for the treatment of CLDs. Stem Cells Translational Medicine *2017;6:272–284*

**Significance Statement**

This is the first report that the transplantation of human menstrual blood‐derived stem cells (MenSCs) has a therapeutic effect on liver fibrosis in a mouse model. Analysis of α‐smooth muscle actin and transforming growth factor‐β1 expression found that MenSCs suppressed the activated hepatic stellate cells (HSCs). Furthermore, the indirect coculture with LX‐2 cells (activated HSCs) showed that MenSCs targeted HSCs through secretion of paracrine mediators (cytokines), namely, monocyte chemotactic protein‐1, interleukin‐6, hepatocyte growth factor, growth‐related oncogene, interleukin‐8, and osteoprotegerin. These findings show that MenSCs could be used as a novel therapeutic option for the treatment of chronic liver diseases.

## Introduction

Liver fibrosis occurs at the final stages of various chronic liver diseases and causes a huge burden with high rates of morbidity and mortality worldwide 1, 2. Fibrosis is a reversible wound‐healing response characterized by the accumulation of extracellular matrix (ECM) proteins at sites of liver injury 3, 4. Currently, orthotopic liver transplantation is the most effective therapy for reversing fibrotic liver; however, its application is restricted because of organ donor shortage, surgical complications, and the requirement for lifelong immunosuppression 5, 6. Therefore, alternative therapeutic strategies to treat liver fibrosis are urgently needed.

Mesenchymal stem cell (MSC)‐based therapy may be an attractive option to treat liver fibrosis 6 7 8. The bone marrow (BM) is the most common source of MSCs, and BM‐MSCs have been extensively studied in animal models and clinical trials to evaluate their therapeutic effects 5, 9 10 11 12 13, however, the isolation of BM‐MSCs is a painful and invasive procedure to donors 8, 14. Adipose tissue (AT) is another common source with larger quantities of progenitor cells compared with the BM, and AT‐MSCs have been studied in some animal models for treating liver diseases 15 16 17 18. Currently, not all animal experiments and clinical trials have shown positive effects of BM‐MSCs in treating liver fibrosis 8, 19 20 21. This study focuses study on an alternative MSC source.

Human menstrual blood‐derived stem cells (MenSCs), also known as menstrual blood‐derived mesenchymal stem cells, are isolated from the menstrual fluid 22 23 24. MenSCs have phenotypes and properties similar to BM‐MSCs, including a spindle shape, classical three‐lineage differentiation, and surface marker expression 22, 24 25 26. Compared with BM‐MSCs, MenSCs exhibit a higher proliferation rate and can be obtained through a simple, safe, painless procedure that is not ethically controversial 25, 27 28 29 30. Moreover, our group and others have reported that MenSCs possess low immunogenicity and can be stably expanded for at least 20 passages without acquiring genetic abnormalities, emphasizing the convenience of their use 27, 31, 32. The therapeutic potential of MenSCs has been demonstrated in several disease models, such as Duchenne muscular dystrophy, stroke, type 1 diabetes, premature ovarian failure, and myocardial infarction 31, 33 34 35 36 37 38, further supporting that MenSC‐based therapy may have future clinical applications. While the capacity for MenSCs to differentiate into functional hepatocyte‐like cells has been previously described 39, 40, little is known about their therapeutic potential in liver fibrosis.

In the present study, the therapeutic effects of MenSCs were evaluated following transplantation into a carbon tetrachloride (CCl_4_)‐induced mouse model of liver fibrosis in vivo. Moreover, the functional role of MenSCs in this process was examined by MenSC/hepatic stellate cell (hereafter termed stellate cells) coculture experiments in vitro.

## Materials and Methods

### Animals and Cells

Male imprinting control region (ICR) mice (6–8 weeks) were purchased from the Shanghai Laboratory Animal Center (Shanghai, China, http://english.sibs.cas.cn/rs/fs/ShanghaiLaboratoryAnimalCenterCAS) and were housed under standard conditions with 12‐hour light/dark cycle at the Laboratory Animal Center of Zhejiang University. Food and water were available ad libitum. All animal experiments were approved by the Animal Care and Use Committees of Zhejiang University.

MenSCs were isolated and maintained as described previously 22, 31. The isolation procedure and informed consent form signed by volunteer donors were both approved by the Ethics Committee of Zhejiang University. Briefly, menstrual blood samples were collected with a DivaCup (Diva International, Kitchener, ON, Canada, http://divacup.com) from four healthy, menstruating women; cells from these donors showed no significant interdonor variability in a previous study 31. Mononuclear cells were separated by density gradient centrifugation with Ficoll‐Paque (Thermo Fisher Scientific Life Sciences, Oakwood Village, OH, https://www.thermofisher.com). The interlayer cells were then collected and cultured in Chang medium (S‐Evans Biosciences, Hangzhou, China, http://www.sebio.net.cn) at 37°C in a 5% CO_2_ humidified atmosphere with medium changes every 2–3 days. Cells were subcultured in tissue culture flasks (Corning, Corning, NY, http://www.corning.com) by using 0.25% trypsin‐EDTA (Thermo Fisher Scientific Life Sciences) upon reaching 80%–90% confluence. All MenSCs (including green fluorescent protein [GFP]‐transduced MenSCs) were used in experiments before passage 8.

LX‐2 is an immortalized stellate cell line commonly used to mimic activated stellate cells in vitro 41. The LX‐2 cells were provided as a generous gift from Zhiliang Gao 11 (Third Affiliated Hospital, Sun Yat‐sen University, Guangdong, China, http://www.sysu.edu.cn). LX‐2 cells were cultured in low‐glucose Dulbecco's modified Eagle medium (L‐DMEM; GE Life Sciences, Pasching, Austria, http://www.gelifesciences.com) supplemented with 10% fetal bovine serum (FBS; Thermo Fisher Scientific Life Sciences), 100 IU/ml penicillin (Sigma‐Aldrich, St. Louis, MO, http://www.sigmaaldrich.com), and 100 μg/ml streptomycin (Sigma‐Aldrich).

### CCl_4_‐Induced Liver Fibrosis and MenSC Transplantation

To induce liver fibrosis, ICR mice (20 ± 2 g) were intraperitoneally injected with CCl_4_ (Sigma‐Aldrich) twice a week for 4 weeks (*n* = 6; CCl_4_ group). The dose of CCl_4_ (dissolved in olive oil at 10% solution) was 1 milliliter per kilogram of body weight. No animals died during the experiments after CCl_4_ administration. Mice were injected an equal volume of olive oil alone as a vehicle control for the liver fibrosis model (*n* = 6; vehicle group).

To evaluate the therapeutic efficacy of MenSCs on liver fibrosis, 5 × 10^5^ cells in 1 ml of phosphate‐buffered saline (PBS) were injected into the tail vein of mice in the liver fibrosis model after 4 weeks of CCl_4_ treatment; the group of mice with MenSC transplantation was considered the MenSC group. Three cell samples were randomly chosen from previous donors 31, which were independent and not pooled. Each of the three donor samples was used to inject two mice with MenSCs in the MenSC group (*n* = 6). Mice were randomly assigned to groups in the study. Mice injected with an equal amount of PBS without MenSCs were used as the vehicle control for MenSC transplantation (PBS group). Subsequently, animals were anesthetized with sodium pentobarbital (50 mg/kg; Solarbio Bioscience and Technology, Shanghai, China, http://www.shsolarbio.com) at 1 week (1W) or 2 weeks (2W) after MenSC transplantation/PBS injection. CCl_4_ administration was continued during the 2‐week treatment period in the MenSC and PBS groups to prevent spontaneous recovery of liver fibrosis. A schematic diagram showing the time course of the animal experiment is shown in supplemental online Figure 1. Serum and liver tissue samples from all four groups (vehicle, CCl_4_, PBS 1W/2W, and MenSC 1W/2W) were collected and stored at −80°C.

### Lentiviral Transduction, MenSC Immunophenotyping, and Cell Tracking

The lentiviral GFP expression vector pHBLV‐IRES‐ZsGreen‐PGK‐puro was purchased from Hanbio (Shanghai, China, http://www.hanbio.net). Lentivirus‐containing supernatants were filtered through a 0.22‐μm cellulose acetate membrane filter (EMD Millipore, Billerica, MA, http://www.emdmillipore.com), and the viral titer was calculated to be 10^8^ transducing units per milliliter. Viral supernatants were then used to infect MenSCs at passage 2–3 at a multiplicity of infection of 50 in the presence of 6 μg/ml polybrene (Sigma‐Aldrich) for 24 hours. Subsequently, infected cells were selected with puromycin (3 μg/ml; Sigma‐Aldrich) for 2 weeks.

For GFP^+^ MenSC immunophenotyping, the expression of cell surface markers was evaluated by fluorescence‐activated cell sorting with nontransduced MenSCs as a control. Briefly, 5 × 10^5^ cells were collected and washed twice with stain buffer (BD Biosciences, San Jose, CA, http://www.bdbiosciences.com) before a 20‐minute incubation in the dark with antibodies targeting various cell surface markers, including CD29, CD34, CD45, CD73, CD90, CD105, CD117, and human leukocyte antigen‐DR (HLA‐DR). Stained cells were resuspended in the stain buffer and analyzed with a FC500 flow cytometer (Beckman Coulter, Miami, FL, https://www.beckmancoulter.com). IgG1 was used as an isotype control for the anti‐CD29, anti‐CD34, anti‐CD45, anti‐CD73, anti‐CD90, anti‐CD105, and anti‐CD117 antibodies, whereas IgG2a served as an isotype control for the anti‐HLA‐DR antibody. Detailed information on the antibodies is included in supplemental online Table 1.

For cell tracking experiments, 5 × 10^5^ GFP‐expressing MenSCs in 1 ml of PBS were transplanted into mice with CCl_4_ injury (twice a week for 4 weeks; supplemental online Fig. 1). Mice were euthanized at 1 or 2 weeks (GFP^+^ MenSC 1W/2W) after cell transplantation, and liver tissues were harvested and rapidly frozen. Nontransduced MenSCs served as a negative control.

### MenSC/LX‐2 Cocultures

A coculture Transwell chamber (24‐mm diameter, 0.4‐μm pore size; Corning) was used to assess the effects of MenSCs on LX‐2 cells in vitro. LX‐2 cells were seeded into the lower chamber in 1.8 ml of L‐DMEM with 10% FBS, and MenSCs were seeded in the upper compartment in 1.2 ml of the same medium. The three groups were as follows: LX‐2 group (*n* = 4), 2 × 10^5^ LX‐2 cells seeded in the lower chamber; MenSC group (*n* = 4), 2 × 10^5^ MenSCs seeded in the upper chamber; and coculture group (*n* = 4), 2 × 10^5^ LX‐2 cells and 2 × 10^5^ MenSCs seeded in the lower and upper chambers, respectively. Samples were collected after culturing for 24, 48, or 72 hours.

### Liver Function Tests and Histological Analysis

Liver function was assessed by analyzing serum alanine aminotransferase (ALT), aspartate aminotransferase (AST), albumin (ALB), alkaline phosphatase (ALP), and total bilirubin (TBIL) levels using commercial kits according to the manufacturer's instructions (Nanjing Jiancheng Bioengineering Institute, Nanjing, China, http://www.njjcbio.com).

To analyze collagen content, hepatic tissues were fixed in 10% formalin overnight at 4°C, embedded in paraffin, and sectioned at 5‐μm thickness with a Microm HM325 rotary microtome (Thermo Fisher Scientific Life Sciences). Sections were stained with 0.1% Sirius red (Sigma‐Aldrich) following the manufacturer's protocol and then imaged with an Olympus IX83 inverted microscope (Olympus, Tokyo, Japan, http://www.olympus.co.jp) using Olympus cellSens software (cellSens Standard 1.9). The fibrotic area (collagen area/total area) was quantified with Image Pro Plus 6 software (Media Cybernetics, Rockville, MD, http://www.mediacy.com) on five random, nonoverlapping fields from each sample.

### Immunohistochemistry, Immunofluorescence, and Quantitative Real‐Time Reverse Transcription‐Polymerase Chain Reaction

Immunohistochemistry was performed to assess α‐smooth muscle actin (α‐SMA) and transforming growth factor‐β 1 (TGF‐β1) using a Dako EnVision IHC system (Dako, Denmark) according to the manufacturer's procedures. Briefly, 5‐μm paraffin sections were deparaffinized with xylene and then rehydrated through descending grades of alcohol. Antigen retrieval was performed by heating in 10 mM sodium citrate buffer (pH 6.0) for 20 minutes and incubating in 3% hydrogen peroxide for 10 minutes to block endogenous peroxidases. Tissue sections were subsequently incubated with primary antibodies overnight at 4°C, followed by the appropriate horseradish peroxidase‐conjugated secondary antibodies (Dako EnVision System; Dako) for 30 minutes. Immunoreactive areas were visualized with diaminobenzidine (Dako) solution for 20 minutes. Slides were counterstained with hematoxylin before analysis.

Immunofluorescence analysis was performed as previously described 31. Briefly, frozen tissues collected from GFP^+^ and control MenSC‐transplanted mice were embedded in Tissue‐Tek O.C.T. compound (Sakura Finetek, Torrance, CA, http://www.sakura-americas.com), sectioned (5‐μm thickness), and incubated with antihuman cytokeratin 18 (CK‐18) antibodies overnight at 4°C followed by incubation with Cy3 affinipure goat antimouse IgG (EarthOx Life Sciences, Millbrae, CA, http://www.earthox.net) for 1 hour at room temperature to examine MenSC differentiation into hepatocyte‐like cells. Nuclei were then counterstained with 4′,6‐diamidino‐2‐phenylindole (Sigma‐Aldrich) for 20 minutes. Sections were imaged with an IX83 microscope equipped with a U‐HGLGPS fluorescence illumination source (Olympus).

Liver tissues were homogenized in 1 ml of RNAiso Plus (TaKaRa Bio Inc., Shiga, Japan, http://www.takara-bio.co.jp) to isolate total RNA according to the manufacturer's instructions. RNA was then reverse‐transcribed into cDNA by using a FastQuant RT kit (Tiangen Biotech, Beijing, China, http://www.tiangen.com/en). Quantitative real‐time reverse transcription‐polymerase chain reaction (qRT‐PCR) was performed to examine mRNA expression using a CFX96 Multicolor Real‐time PCR Detection System (Bio‐Rad, Hercules, CA, http://www.bio-rad.com) according to a previously described protocol 42. Briefly, reaction mixtures consisted of 1 μl of DNA (10 ng), 8.2 μl of nuclease‐free water, 10 μl of Kapa SYBR Fast qPCR Master Mix (Kapa Biosystems, Wilmington, MA, https://www.kapabiosystems.com), and 0.4 μl of each gene‐specific primer (10 mM). The threshold cycle (CT) value was calculated by using the 2^–ΔΔCT^ method. β‐actin served as an internal control. The primers are described in supplemental online Table 2.

### Cell Proliferation and Cell Cycle Analysis

A Cell Counting Kit‐8 (CCK‐8; Dojindo Molecular Technologies, Rockville, MD, http://www.dojindo.com) was used to evaluate the proliferation of LX‐2 cells in coculture experiments. After culturing for 24, 48, and 72 hours, the CCK‐8 reagent was added to the lower chamber containing LX‐2 cells in the LX‐2 group and the coculture group and incubated for 2 hours according to the manufacturer's protocol. Optical densities at 450 nm (OD_450_) were then measured by using a multifunctional microplate reader (SpectraMax M5; Molecular Devices, Sunnyvale, CA, http://www.moleculardevices.com). For cell cycle analysis, LX‐2 cells were fixed in ice‐cold 70% ethanol at −20°C overnight, centrifuged, and then stained with propidium iodide (Sigma‐Aldrich) in the dark for 30 minutes at 37°C by using a Cell Cycle and Apoptosis Analysis Kit (Beyotime Biotechnology, Haimen, China, https://www.beyotime.com); apoptosis was not evaluated. Cell cycle distributions (G_0_/G_1_, S, and G_2_/M) were then analyzed by flow cytometry. Data are shown as the means ± SDs of four independent biological samples in each group.

### Antibody Arrays and Enzyme‐Linked Immunosorbent Assays

Human cytokine G1000 arrays (AAH‐CYT‐G1000; RayBiotech, Norcross, GA, http://www.raybiotech.com) were used to measure the expression of 120 cytokines ( supplemental online Fig. 2) in culture supernatants at 72 hours after MenSC/LX‐2 coculturing, according to the manufacturer's instructions. From each group (*n* = 4), the culture supernatants from the lower and upper chambers were mixed and harvested after culturing for 72 hours. Positive signals were captured on glass chips by using a laser scanner (GenePix 4000B Microarray Scanner; Molecular Devices), and the fluorescent intensities were normalized to those of internal positive controls. These cytokines were screened by the following integrated conditions: coculture group compared with LX‐2 cells (*p* < .05), coculture group compared with MenSCs (*p* < .05), and fluorescence intensity values exceeding 300 (a relative value; recommended by RayBiotech). Differentially expressed proteins were arranged by hierarchical clustering and represented as a heat map. The heat map was generated by using R software ( http://www.r-project.org).

Based on the antibody array results, we examined protein levels of monocyte chemoattractant protein‐1 (MCP‐1), interleukin‐6 (IL‐6), hepatocyte growth factor (HGF), growth‐related oncogene (GRO), IL‐8, and osteoprotegerin (OPG) using enzyme‐linked immunosorbent assay (ELISA) kits (RayBiotech) both in culture supernatants and cell lysates, according to a previously described protocol 43. For ELISAs using culture supernatants, the culture supernatants of three groups (LX‐2 cells, MenSCs, and coculture group) from the lower and upper chambers were mixed and harvested after culturing for 72 hours. For ELISAs using cell lysates, cell samples were collected from each group (i.e., the LX‐2 group [LX‐2 cells collected from the lower chamber, named as LX‐2], the MenSC group [MenSCs collected from the upper chamber, named as MenSC], and the coculture group [LX‐2 cells collected from the lower chamber and MenSCs collected from the upper chamber, named as coculture‐LX‐2 and coculture‐MenSC, respectively]), after culturing for 72 hours. Cells were lysed in lysis buffer (Cell Signaling Technology, Beverly, MA, http://www.cellsignal.com). OD_450_ values were measured by using a microplate reader. Four independent samples were measured, and data are shown as the mean ± SDs, recorded as picograms per milliliter for culture supernatants or picograms per milligram for cell lysates.

### Gene Ontology and Pathway Analyses

Differentially expressed proteins identified in the antibody arrays ( supplemental online Table 3) were analyzed with Gene Ontology (GO) (AmiGO 2; http://amigo.geneontology.org) and KEGG Orthology Based Annotation System pathway ( http://kobas.cbi.pku.edu.cn) analyses. The top 10 canonical annotations and pathways were presented.

### Statistical Analysis

Statistical analysis was performed in SPSS 16.0 (IBM, Armonk, NY, http://www.ibm.com). All data represent the means ± SDs for at least three independent samples. The data were subjected to one‐way analysis of variance followed by Student's *t* tests. Differences with *p* values of less than .05 were considered statistically significant.

## Results

### Morphology and Immunophenotyping of MenSCs

**Figure 1:**
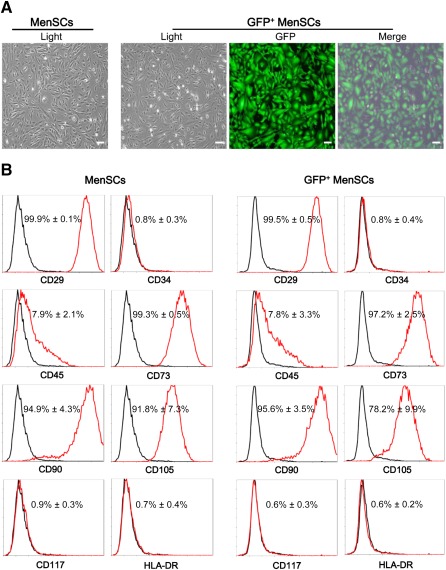
Characterization of cell morphology and marker in MenSCs and GFP^+^ MenSCs. **(A):** Representative images of MenSCs and GFP^+^ MenSCs. Scale bars = 100 μm. **(B):** Characterization of cell surface markers in MenSCs and GFP^+^ MenSCs (red) and in isotype controls (black). Data represent the means ± SDs (*n* = 4). There was no significant difference of these markers in MenSCs and GFP^+^ MenSCs (*p* > .05). Abbreviations: GFP, green fluorescent protein; HLA‐DR, human leukocyte antigen‐DR; MenSCs, menstrual blood‐derived stem cells.

Similar to MSCs, GFP‐positive and nontransduced MenSCs exhibited a spindle‐shaped, fibroblast‐like morphology (Fig. 1A); strong CD29, CD73, CD90, and CD105 expression; and weak CD45 expression; and were negative for CD34, CD117, and HLA‐DR (Fig. 1B). Additionally, more than 99% of infected cells were positive for GFP expression after puromycin selection (Fig. 1A and supplemental online Fig. 3).

### MenSC Transplantation Decreased Collagen Content and improved Liver Function in CCl_4_‐Induced Mice

**Figure 2:**
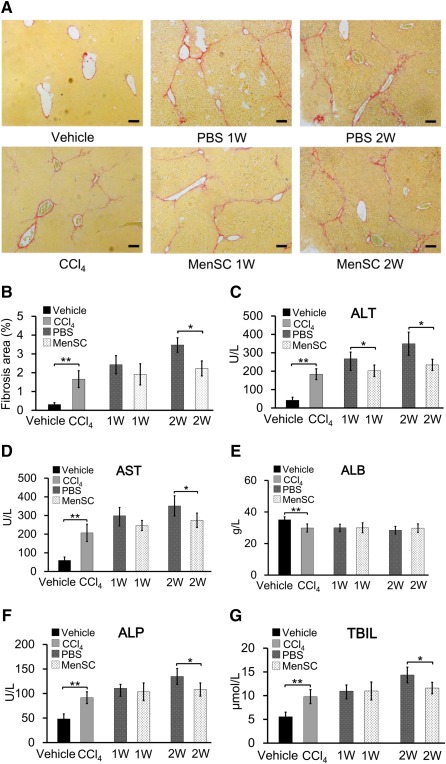
Physiological and pathological indexes in different experimental groups. **(A):** Representative images of liver sections stained with Sirius red to detect collagen proportion. Scale bars = 100 μm. **(B):** Quantitative analysis of fibrosis in different experimental groups. Staining was quantified in five random, nonoverlapping fields. **(C–G):** Liver function tests in the different groups: ALT **(C)**, AST **(D)**, ALB **(E)**, ALP **(F)**, and TBIL **(G)**. Data represent the means ± SDs (*n* = 6). ∗, *p* < .05; ∗∗, *p* < .01. Abbreviations: ALB, albumin; ALP, alkaline phosphatase; ALT, aminotransferase; AST, aspartate aminotransferase; MenSC, menstrual blood‐derived stem cell; PBS, phosphate‐buffered saline; TBIL, total bilirubin; W, week.

In this study, we used the CCl_4_‐induced mouse model of liver fibrosis to investigate whether MenSCs provided therapeutic effects in liver fibrosis. The transplantation protocol is illustrated in supplemental online Figure 1. Fibrosis in harvested livers was assessed by assaying for collagen with Sirius red staining (Fig. 2A, 2B). In the vehicle group, collagen was mostly observed around the central veins and periportal area, and its amount significantly increased after 4 weeks of CCl_4_ injection (Fig. 2A). When quantified, the collagen proportion was slightly lower in the MenSC 1W group than in the PBS 1W group; however, the difference was not significant. In contrast, a significant decrease was noted in the MenSC 2W group (2.2% ± 0.4%) compared with the PBS 2W group (3.5% ± 0.4%; Fig. 2B).

At 1 or 2 weeks after transplantation, the serum levels of ALT, AST, ALB, ALP, and TBIL were assayed (Fig. 2C–2G). Compared with those in the vehicle control group, the values of ALT, AST, ALB, ALP, and TBIL were significantly higher in CCl_4_‐treated mice. Because mice were treated with CCl_4_ continuously throughout the experiment, the serum levels of these liver enzymes progressively increased in the PBS (PBS 1W/2W) and MenSC (MenSC 1W/2W) groups. Only serum ALT was significantly lower in MenSC 1W mice compared with that in their PBS 1W counterparts, whereas ALT, AST, ALP, and TBIL levels were significantly lower in MenSC 2W mice than in PBS 2W mice. Notably, the expression of ALB was not significantly different between MenSC 2W and PBS 2W.

### Migration of MenSCs and α‐SMA/TGF‐β1 Expression After MenSC Transplantation

**Figure 3:**
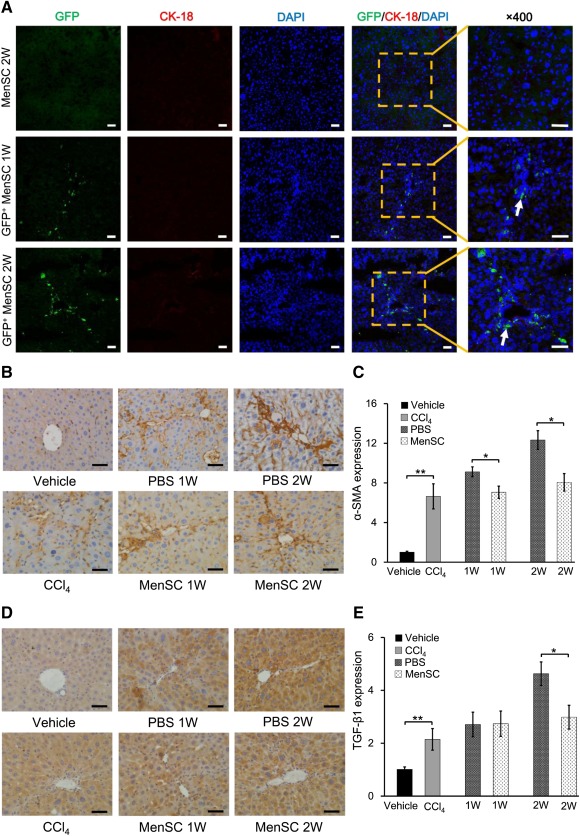
Cell tracing and gene expressing in different experimental groups. **(A):** MenSC localization and CK‐18 expression examined in the fibrotic livers of mice at 1 and 2 weeks after transplantation with GFP‐positive (GFP^+^ MenSC 1W and 2W) or unlabeled MenSCs (MenSC 2W). GFP (green), CK‐18 (red), and DAPI (blue) are shown. White arrows (magnification, ×400) indicate fibrotic areas. **(B–E):** The expression of α‐SMA (B, C) and TGF‐β1 (D, E) examined in fibrotic livers by immunohistochemistry and quantitative reverse‐transcriptase polymerase chain reaction analyses. Data represent the means ± SDs (*n* = 6). ∗, *p* < .05; ∗∗, *p* < .01. Scale bars = 50 μm. Abbreviations: CK‐18, cytokeratin 18; DAPI, 4′,6‐diamidino‐2‐phenylindole; GFP, green fluorescent protein; MenSC, menstrual blood‐derived stem cell; PBS, phosphate‐buffered saline; α‐SMA, α‐smooth muscle actin; TGF‐β1, transforming growth factor‐β1; W, week.

The migration of stem cells into the site of injury is an important precondition for injury repair. To verify whether MenSCs migrated to the sites of liver injury, the localization of GFP^+^ MenSCs was analyzed at 1 and 2 weeks after transplantation (Fig. 3A). Notably, GFP^+^ cells predominantly localized to injured sites around fibrotic or portal areas (white arrows at ×400 magnification). Additionally, to observe the fate of the transplanted cells, we examined the distribution of GFP^+^ MenSCs in major tissues (including the liver, lung, spleen, kidney, and heart) at 1 and 2 weeks (GFP^+^ 1W and GFP^+^ 2W, respectively) after transplantation in normal mice (NM) and liver fibrotic mice (LFM; supplemental online Figure 4). As shown in supplemental online Figure 4A, MenSCs were distributed to the liver in NM both in GFP^+^ 1W and GFP^+^ 2W. Compared with that in NM livers, MenSCs in LFM livers were markedly increased ( supplemental online Fig. 4B; *p* < .05 in GFP^+^ 1W and *p* < .01 in GFP^+^ 2W). More importantly, MenSCs had dominantly migrated to the liver both in GFP^+^ 1W and GFP^+^ 2W (particularly obvious for GFP^+^ 2W) in LFM. These results indicated that the homing was specific to the injury site ( supplemental online Fig. 4). Furthermore, CK‐18 staining was used to monitor whether transplanted MenSCs differentiated into functional hepatocyte‐like cells, revealing that MenSCs did not acquire CK‐18 expression at 1 or 2 weeks after transplantation (Fig. 3A).

α‐SMA and TGF‐β1 are important factors for activation of stellate cells. To examine the effects of MenSC transplantation on inhibiting stellate cells in the liver, immunohistochemical staining and qRT‐PCR analysis were used to examine α‐SMA and TGF‐β1 expression in liver tissues after MenSC transplantation. As expected, α‐SMA expression in the vehicle group was restricted to the walls of the central veins and vascular smooth muscle cells; however, CCl_4_ treatment resulted in perisinusoidal α‐SMA expression, indicative of stellate cell activation in the affected lobuli (Fig. 3B). Notably, this staining was decreased in MenSC 1W and MenSC 2W groups when compared with that in the PBS 1W and PBS 2W controls, respectively (Fig. 3B). These findings were also confirmed by qRT‐PCR analysis, which revealed a significant upregulation of α‐SMA (6.6‐fold) in the CCl_4_ group compared with that in vehicle controls. Moreover, the expression was significantly lower in MenSC 1W (7.1‐fold) and MenSC 2W (8.1‐fold) groups than those in PBS 1W (9.1‐fold) and PBS 2W (12.3‐fold) controls, respectively (Fig. 3C). These results were further validated by Western blot analysis ( supplemental online Fig. 5). TGF‐β1 expression was clearly observed throughout the liver (Fig. 3D). Notably, TGF‐β1 expression was weakly detected in the vehicle group, whereas more marked expression was found in the CCl_4_ group. Although no obvious differences were observed between MenSC 1W and PBS 1W groups, cytoplasmic TGF‐β1 expression in the PBS 2W group was more intense than that observed in the MenSC 2W group (Fig. 3D). The relative TGF‐β1 expression in the CCl_4_ group (2.1‐fold) was markedly increased over that in the vehicle group; furthermore, although no significant differences in TGF‐β1 expression were observed between the MenSC 1W and PBS 1W groups, the expression of TGF‐β in the MenSC 2W group (threefold) was obviously decreased when compared with that in the PBS 2W group (4.6‐fold; Fig. 3E).

### MenSCs Suppressed LX‐2 Cell Proliferation by Inducing G_0_/G_1_‐Phase Arrest

**Figure 4:**
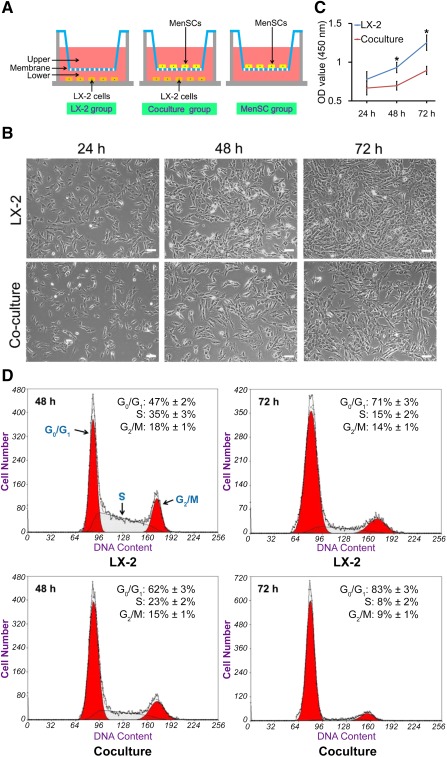
The inhibitory effects of MenSCs on LX‐2 cell proliferation in single cultures and cocultures. **(A):** Experimental design for MenSC and LX‐2 coculture experiments. **(B):** Representative images of LX‐2 cells in single cultures and cocultures at 24, 48, and 72 hours. Scale bar = 100 μm. **(C):** The inhibitory effects of MenSCs on LX‐2 cell proliferation examined using CCK‐8 assays in single cultures and cocultures at 24, 48, and 72 hours. **(D):** LX‐2 cell cycle distribution after growth in single cultures or cocultures for 48 or 72 hours. The percentage of G_0_/G_1_‐phase LX‐2 cells significantly higher in the cocultured cells than in the single‐cell controls at 48 and 72 hours. G_0_/G_1_, S, and G_2_/M phases are marked with arrows. Abbreviations: MenSC, menstrual blood‐derived stem cell; OD, optical density.

To further investigate the effects of MenSC transplantation on the inhibition of stellate cells, transwell coculture was used to determine the effects of MenSCs on stellate cell proliferation (Fig. 4A). The activated state of LX‐2 cells was defined as greater than 95% α‐SMA positive ( supplemental online Fig. 6). The amount of growing cells was visualized under an inverted microscope (Fig. 4B). At 24 hours, the number of LX‐2 cells in the coculture group was nearly same as that in the LX‐2 cells; however, the number of cocultured LX‐2 cells was obviously decreased at 48 and 72 hours compared with that in the LX‐2‐only culture (data not shown). CCK‐8 assays were performed to evaluate cell proliferation. As expected, although there were no significant differences between single and cocultured LX‐2 cells at 24 hours, the proliferative capability of LX‐2 cells in the coculture group was significantly decreased at 48 hours (OD_450_, 0.7 ± 0.1) and 72 hours (0.9 ± 0.1) when compared with that in the LX‐2 group at 48 hours (0.9 ± 0.1) and 72 hours (1.3 ± 0.1) (Fig. 4C). Next, we examined cell cycle progression in LX‐2 cells in both single cultures and cocultures at 48 and 72 hours by flow cytometry (Fig. 4D). These analyses revealed that the percentage of G_0_/G_1_‐phase LX‐2 cells was significantly higher in the cocultured cells than in the single‐cell type controls at 48 and 72 hours. Detailed analysis of the G_0_/G_1_ phase is shown in supplemental online Figure 7.

### Differential Cytokine Expression in LX‐2/MenSC Cocultures

**Figure 5:**
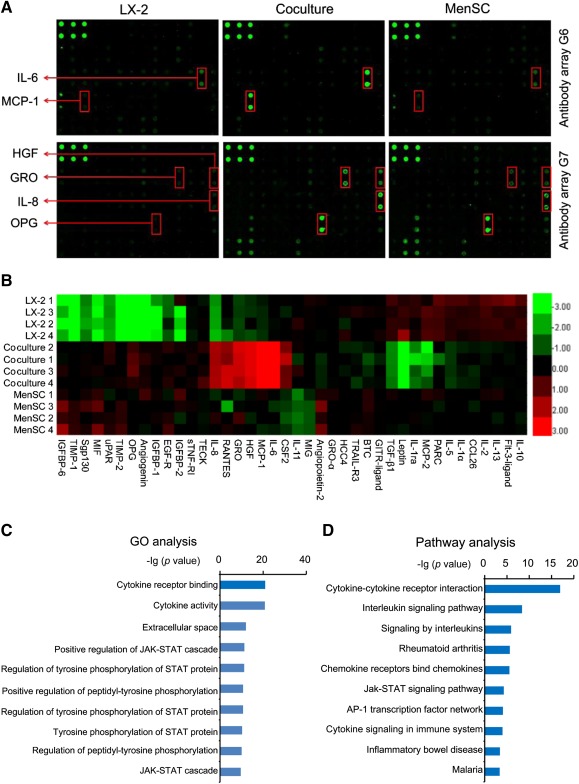
Cytokine secretion in MenSC/LX‐2 cocultures. **(A):** Representative array images are shown (*n* = 4). Cytokines of interest (MCP‐1, IL‐6, HGF, GRO, IL‐8, and OPG) are highlighted with red boxes and arrows, accordingly. **(B):** Differential cytokine expression shown as a heat map. Red, green, and black colors represent upregulated, downregulated, and unchanged expression, respectively. **(C, D):** Results from GO **(C)** and pathway **(D)** bioinformatic analyses. The top 10 pathways are shown with their associated *p* values. Abbreviations: GO, Gene Ontology; GRO, growth‐related oncogene; HGF, hepatocyte growth factor; IL‐6, interleukin 6; MCP‐1, monocyte chemoattractant protein‐1; MenSC, menstrual blood‐derived stem cell; OPG, osteoprotegerin.

To identify molecules involved in mediating the suppressive effects of MenSCs on LX‐2 cell proliferation, we used an antibody array to examine cytokine levels in the supernatants of the LX‐2 cells, MenSCs, and coculture group (Fig. 4A). As shown in Figure 5A, several cytokines (i.e., MCP‐1, IL‐6, HGF, GRO, IL‐8, and OPG) were highly expressed in the coculture group compared with those in the LX‐2 and MenSC single‐culture control groups. Among 120 cytokines evaluated ( supplemental online Fig. 2), some were not detectably expressed or showed extremely low expression (data not shown). Differentially expressed cytokines were visualized by using a heat map (Fig. 5B); the average fluorescence intensities are shown in supplemental online Figure 8. Importantly, the secretion of MCP‐1 and IL‐6 was significantly increased in the coculture group compared with those in the LX‐2 and MenSC control groups. Moreover, secretion of HGF, GRO, and IL‐8 was also moderately enhanced. Interestingly, OPG expression was extremely high in the coculture and MenSCs, but was low in the LX‐2 cells (Fig. 5A, 5B; supplemental online Fig. 8). In contrast to the above cytokines, secretion of leptin, TGF‐β1, and MCP‐2 was markedly lower in cocultures than in cultures of LX‐2 cells or MenSCs alone. GO and pathway analyses were subsequently used to identify the top 10 potential pathways that may mediate this process based on these differences in cytokine secretion, and the cytokine‐cytokine receptor interaction pathway was returned as the first hit ( supplemental online Tables 3–5; Fig. 5C, 5D).

**Figure 6:**
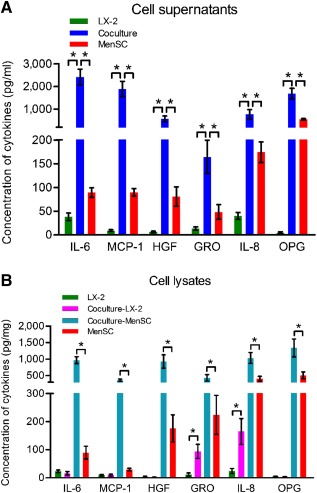
The concentrations of IL‐6, MCP‐1, HGF, GRO, IL‐8, and OPG in culture supernatants **(A)** and cell lysates **(B)** determined by enzyme‐linked immunosorbent assay. Data represent the means ± SDs (*n* = 4). ∗, *p* < .05. Abbreviations: GRO, growth‐related oncogene; HGF, hepatocyte growth factor; IL‐6, interleukin 6; MCP‐1, monocyte chemoattractant protein‐1; MenSC, menstrual blood‐derived stem cell; OPG, osteoprotegerin.

Because antibody arrays are semiquantitative, we performed ELISA to accurately quantify the concentrations of MCP‐1, IL‐6, HGF, GRO, IL‐8, and OPG in both culture supernatants (Fig. 6A) and cell lysates (Fig. 6B). Consistent with the findings in antibody arrays, the protein levels of all six cytokines were significantly increased in the coculture group of culture supernatants (Fig. 6A). Furthermore, to verify that these factors were primarily secreted by MenSCs, the concentrations of cell lysates from all three cultures (with four cell groups of LX‐2 cells, coculture‐LX‐2 cells, coculture‐MenSCs, and MenSCs) were also examined by ELISA (Figs. 4A, 6B). Importantly, the concentrations of MCP‐1 (361 ± 33 versus 29 ± 5 pg/mg; 12‐fold), IL‐6 (967 ± 104 versus 90 ± 23 pg/mg; 11‐fold), HGF (927 ± 204 versus 176 ± 48 pg/mg; fivefold), GRO (432 ± 96 versus 224 ± 69 pg/mg; twofold), IL‐8 (1033 ± 163 versus 403 ± 75 pg/mg; threefold), and OPG (1338 ± 270 versus 510 ± 103 pg/mg; threefold) were obviously enhanced in coculture MenSCs compared with those in the single MenSC culture. Likewise, the concentrations of GRO (94 ± 25 versus 12 ± 6 pg/mg; eightfold) and IL‐8 (165 ± 46 versus 24 ± 9 pg/mg; sevenfold) were significantly increased in coculture LX‐2 cells compared with those in single LX‐2 cultures.

## Discussion

Menstrual fluid has important advantages over other cell sources for the collection and isolation of MSCs, including simple, repeated, noninvasive sampling 25. Moreover, there is a growing need for better cell sources providing multipotent, highly proliferative cells sufficient for therapeutic doses at lower passage numbers 25, 27, 31. Liver fibrosis is a major public health issue for which there are no effective therapeutic strategies 4, 44, and MSCs have been shown to improve liver fibrosis in preclinical mouse and rat models and in recent clinical trials 5, 14, 45 46 47 48. However, some reports have also shown that these cells may act as a double‐edged sword by spontaneously undergoing malignant transformation 19, 21, 49, 50. In the present study, MenSCs significantly reduced collagen deposition and improved liver function within 2 weeks of transplantation in a mouse model of liver fibrosis. Furthermore, MenSCs migrated to sites of liver injury around the fibrotic area.

Several mechanisms have been proposed to explain the therapeutic effects of MSCs on liver fibrosis, including hepatocyte differentiation/regeneration, immunomodulation, and inhibition of activated stellate cell proliferation, particularly through a paracrine mechanism 5, 7, 14, 45, 51. Although our group and Khanjani et al. have reported that MenSCs can effectively differentiate into functional hepatocyte‐like cells in vitro 39, 40, the present study suggested that few, if any, MenSCs underwent hepatocyte differentiation based on CK‐18 expression. One explanation for the lack of CK‐18 expression is that the complex in vivo microenvironment may delay MenSC differentiation 52. Our results suggest that MenSC differentiation toward a hepatocyte lineage is not necessary for MenSC‐mediated attenuation of liver fibrosis in this model.

Activated stellate cells are the primary source of myofibroblasts that produce ECM in the liver, particularly during fibrogenesis 53, 54. TGF‐β1, a potent profibrogenic cytokine that plays a critical role in the activation of fibrogenic myofibroblasts 46, 55, triggers stellate cell activation, leading to proliferation and expression of α‐SMA, a classical marker of activated stellate cells 45, 54. In the present study, we found that expression of α‐SMA and TGF‐β1 was significantly lower 2 weeks after transplantation in the livers of mice treated with MenSCs, suggesting that MenSC may ameliorate liver fibrosis in part by reducing expression of profibrogenic factors.

Consistent with this finding, some reports have shown that human MSCs may inhibit LX‐2 cell proliferation in coculture experiments 11, 56. Thus, we used a transwell coculture system to determine whether a similar mechanism could explain our transplantation results. The in vitro assays demonstrated that MenSCs effectively suppress LX‐2 proliferation. Although we did not measure hepatocyte proliferation in the transplantation studies, the potential for reducing proliferation, perhaps by regulating expression of profibrogenic factors, is supported by this in vitro study.

MSCs secrete a wide variety of cytokines that may suppress liver fibrosis through paracrine effects 9, 13, 45, 56, 57. Accordingly, we identified six secreted cytokines (MCP‐1, IL‐6, HGF, GRO, IL‐8, and OPG) in coculture experiments using antibody arrays and ELISAs. Subsequent bioinformatic analysis revealed that cytokine‐cytokine receptor interactions were the most likely physiological mechanism underlying the observed inhibitory effects on LX‐2 cell proliferation.

Generally, IL‐6 serves as a proinflammatory factor that facilitates liver fibrosis by promoting the differentiation of stellate cells into myofibroblasts 58, 59; however, IL‐6 has also been shown to act as a prosurvival factor, stimulating hepatocyte proliferation 60, 61. Alternatively, the increased levels of MCP‐1 observed in cocultures appear to be contradictory to the proposed proinflammatory effects 62, 63, but consistent with recent reports demonstrating the anti‐inflammatory role of MCP‐1 in liver fibrosis 13, 62. Meier et al. recently revealed that MSCs secrete insulin‐like growth factor binding protein‐2, IL‐1 receptor‐α, IL‐6, and MCP‐1, which exert antifibrotic effects in an experimental mouse model of liver fibrosis 13. Similarly, IL‐6 and MCP‐1 were both highly secreted by MenSCs in our study, suggesting that these cytokines likely act as protective factors during the initial phases of fibrogenesis. Many researchers have reported that HGF exhibits therapeutic effects in liver fibrosis by promoting hepatocellular regeneration or inducing the apoptosis of stellate cells 11, 56, 64. We also observed a similar antifibrotic function of MenSC‐secreted HGF by inducing G_0_/G_1_‐phase arrest in stellate cells. Additionally, combined treatment with MSCs and IL‐6/HGF can significantly ameliorate liver fibrosis compared with treatment with MSCs or IL‐6/HGF alone in vivo and in vitro 11, 65. These data suggested that stem cells may have stronger therapeutic efficacy in the presence of adjuvant cytokines and support further investigation into the synergistic roles of MenSCs and cytokines.

Chemokines are important determinants in the pathogenesis and outcomes of hepatic disease. Additionally, chemokines have been shown to influence the fibrogenic effects of local stellate cells and Kupffer cells 66. Unexpectedly, OPG was the only cytokine that was highly expressed specifically in single‐culture MenSC and coculture groups. OPG is a soluble tumor necrosis factor‐related apoptosis‐inducing ligand receptor that is mainly secreted by osteoblasts and has been shown to be associated with the regulation of bone resorption, modeling, and remodeling 67, 68. Additionally, some studies have reported the therapeutic potential of MSCs in some bone‐related diseases, such as osteoporosis, fracture, and rheumatoid arthritis 69 70 71.

Although we demonstrated that MenSCs ameliorated liver fibrosis in mice and explored some secreted cytokines in the present study, there are still many challenges to overcome for application of MenSCs in clinical medicine. For example, many patients with end‐stage liver disease exhibit fibrosis as a major pathology, but lack sufficient hepatocyte abundance to maintain good liver function. Thus, simply reducing fibrosis without replenishing the hepatocyte pool may be insufficient in these patients. Therefore, cell differentiation or other methods are needed to produce a sufficient hepatocyte pool and provide improved therapies in these patients.

## Conclusion

In this report, we present preliminary evidence demonstrating the potential of MenSCs for reducing liver fibrosis. These data suggest that MenSC transplantation deserves further study as an adjunctive or alternative approach to treatment of fibrotic liver diseases.

## Author Contributions

Lijun Chen: conception and design, collection and/or assembly of data, data analysis and interpretation, manuscript writing; C.Z. and Lu Chen: collection and/or assembly of data, data analysis and interpretation, manuscript writing; X. Wang and B.X.: collection and/or assembly of data, data analysis and interpretation; X. Wu and X.M.: administrative support, collection and/or assembly of data, data analysis and interpretation; Y.G., L.Y., and B.C.: provision of study material, data analysis and interpretation; J.W. and C.X.: conception and design, financial support, provision of study material, data analysis and interpretation, manuscript writing, final approval of manuscript.

## Disclosure of Potential Conflicts of Interest

The authors indicated no potential conflicts of interest.

## Supporting information

Supporting Information
