# Supplementary material for: Human Menstrual Blood‐Derived Stem Cells Ameliorate Liver Fibrosis in Mice by Targeting Hepatic Stellate Cells via Paracrine Mediators
Source: Stem Cells Transl Med. 2016 Jul 28;6(1):272–84. doi: 10.5966/sctm.2015-0265 (PMC5442725; doi:10.5966/sctm.2015-0265)
Supplement: Supplementary file 1 — Supporting Information [file SCT3-6-272-s001.pdf]

Supplemental Information - Xiang and Wang et al.

Supplementary materials and methods

Transduction efficiency of GFP^+ MenSCs

To confirm the transduction efficiency, GFP^+ MenSCs were examined by FACS with non-transduced MenSCs as a control. A total of 5×10^5 cells were collected and washed twice with stain buffer (BD Biosciences). These cells were then analyzed with the FL1 channel using a flow cytometer.

Tissue distribution of transplanted MenSCs in normal and model mice by in vivo imaging

ICR mice (20 ± 2 g) were housed under standard conditions with a 12-h light/dark cycle at the Laboratory Animal Center of Zhejiang University (China). Food and water were available *ad libitum*. Some mice were intraperitoneally injected with 1 mL/kg CCl_4 (body weight) twice a week for 4 weeks (CCl_4 group). For controls, some mice were fed without CCl_4 treatment. To observe the fate of the transplanted cells, 5×10^5 GFP^+ MenSCs in 1 mL PBS were injected into the tail vein of normal mice (NM; without CCl_4 treatment) or liver fibrosis mice (LFM; CCl_4 treatment). Subsequently, the animals were anesthetized with sodium pentobarbital (50 mg/kg; Solarbio Bioscience & Technology) at 1 or 2 weeks after transplantation of GFP^+ MenSCs (GFP^+ 1W and GFP^+ 2W, respectively). Major organs, including the liver, lung, spleen, kidney, and heart, were separated by surgical scissors and washed with PBS three times to remove the blood clot. All samples (including normal control

mice, GFP$^+$ 1W and GFP$^+$ 2W in NM, and GFP$^+$ 1W and GFP$^+$ 2W in LFM; n = 6 each) were subjected to *in vivo* imaging using an IVIS SPECTRUM system (Caliper Life Sciences, USA), and data were analyzed using Living Image 4.3.1 software.

Western blot analysis

For western blot analysis, liver tissues were collected and homogenized in RIPA lysis buffer (Beyotime Biotechnology, China) supplemented with 1 mM PMSF (Beyotime Biotechnology). A total of 25 μg protein per lane was separated by electrophoresis using NuPAGE Novex 10% Bis-Tris gels (Life Technologies, USA) and then transferred to 0.45-μm PVDF membranes (Millipore) by semi-dry blotting. Membranes were blocked with 0.5% bovine serum albumin (BSA) for 2 h at room temperature and incubated with primary antibodies at 4°C overnight, followed by incubation with HRP-conjugated secondary antibodies (goat anti-mouse or goat anti-rabbit; Bio-Rad, USA) for 1 h at room temperature. Immunoreactive bands were visualized with enhanced chemiluminescence reagent (Bio-Rad) using a Tanon-4500 digital image system (Tanon Science & Technology, China).

Immunofluorescence of LX-2 cells

Immunofluorescence analysis was performed to confirm the activation of LX-2 cells. LX-2 cells were plated on 12-mm diameter glass coverslips in 24-well plates, cultured for 48 h, and then fixed in pre-chilled PBS containing 4% paraformaldehyde for 15 min at 4°C. Cells were permeabilized with 0.25% Triton X-100 (Sigma) in PBS for 15 min at room temperature,

and non-specific binding was blocked for 30 min with 0.5% BSA (Shanghai Sangon Biotech, China) in PBS containing 0.1% Tween-20 (Amresco, USA). Then, fixed cells were incubated with α-SMA antibody overnight at 4°C, followed by incubation with FITC affinipure goat anti-mouse IgG (EarthOx) for 1 h at room temperature. Nuclei were then counterstained with DAPI for 20 min. Stained cells were captured and digitalized using a fluorescence microscope (Olympus).

Supplemental Tables

Table S1. Antibodies used in the study.

**Antibody name**	**Supplier**	**Catalog No.**	**Usage**	**Dilution/dosage**
α-SMA	Abcam	ab7817	WB, IHC, IF	1:200, 1:100, 1:100
TGF-β1	Abcam	ab92486	IHC	1:100
β-actin	Abcam	ab8227	WB	1:2000
CK-18	Abcam	ab7797	IF	1:100
Goat anti-mouse IgG (H + L) HRP conjugate	Bio-Rad	170-6516	WB	1:3000
Goat anti-rabbit IgG (H + L) HRP conjugate	Bio-Rad	170-6515	WB	1:3000
HRP-conjugated anti-mouse IgG	Dako	K4001	IHC	100 μL
HRP-conjugated anti-rabbit IgG	Dako	K4003	IHC	100 μL
FITC affinipure goat anti-mouse IgG (H+L)	EarthOx	E031210	IF	1:200
Cy3 affinipure goat anti-mouse IgG (H+L)	EarthOx	E031610	IF	1:400
PE mouse anti-human CD29	BD Biosciences	561795	FACS	10 μL

PE mouse anti-human CD34	BD Biosciences	560941	FACS	10 µL
PE mouse anti-human CD45	BD Biosciences	560975	FACS	10 µL
PE mouse anti-human CD73	BD Biosciences	561014	FACS	10 µL
PE mouse anti-human CD90	BD Biosciences	561970	FACS	2.5 µL
PE mouse anti-human CD105	BD Biosciences	560839	FACS	2.5 µL
PE mouse anti-human CD117	BD Biosciences	561682	FACS	2.5 µL
PE mouse anti-human HLA-DR	BD Biosciences	560943	FACS	10 µL
PE mouse IgG1	BD Biosciences	555749	FACS	10 µL
PE mouse IgG2a	BD Biosciences	555574	FACS	10 µL

Note: The dilution of antibody follows the instruction. WB, western blot; IHC, immunohistochemistry; IF, immunofluorescence; FACS, fluorescence-activated cell sorting.

Table S2. Primers used for qRT-PCR analysis.

Gene name	Sequence (5'→3')	Fragment size (bp)	Accession No.
β-actin-F	GATGACCCAGATCATGTTTGA	161 bp	NM_007393
β-actin-R	GGAGAGCATAGCCCTCGTAG		
α-SMA-F	GTCCCAGACATCAGGGAGTAA	102 bp	NM_007392
α-SMA-R	TCGGATACTTCAGCGTCAGGA		
TGF-β1-F	TTGCCCTCTACAACCAACACAA	103 bp	NM_011577
TGF-β1-R	GGCTTGCGACCCACGTAGTA		

Note: F, forward; R, reverse.

Table S3. Differentially expressed proteins and their ID used in the analysis.

Protein name	Gene ID	Protein name	Gene ID	Protein name	Gene ID
Angiogenin	283	Leptin	3952	HGF	3082
CCL26	10344	MCP-1	6347	IGFBP-6	3489
Flt-3 Ligand	2322	MCP-2	6355	IL-11	3589
CSF2	1437	MIG	4283	IL-8	3576
IGFBP-1	3484	PARC	6362	MIF	4282
IGFBP-2	3485	RANTES	6352	OPG	4982
IL-10	3586	TGF-β1	7040	sgp130	3572
IL-13	3596	Angiopoietin-2	285	sTNF-RI	7132
IL-1α	3552	BTC	685	TECK	6370
IL-1ra	3557	EGF-R	1956	TIMP-1	7076
IL-2	3558	GITR-Ligand	8995	TIMP-2	7077
IL-5	3567	GRO/GRO-α	2919	TRAIL-R3	8794
IL-6	3569	HCC-4	6360	uPAR	5329

Table S4. Detailed functional annotation of GO analysis.

GO name	GO ID	Input number	Background number	P-value	-Lg(P-value)
Cytokine receptor binding	GO:0005126	29	230	1.19E-21	20.93
Cytokine activity	GO:0005125	28	215	1.74E-21	20.76
Extracellular space	GO:0005615	41	1158	9.78E-13	12.01
Positive regulation of JAK-STAT cascade	GO:0046427	13	59	5.10E-12	11.29
Regulation of tyrosine phosphorylation of STAT protein	GO:0042531	12	45	6.48E-12	11.19
Positive regulation of peptidyl-tyrosine phosphorylation	GO:0050731	16	137	2.15E-11	10.67
Regulation of tyrosine phosphorylation of STAT protein	GO:0042509	12	52	2.15E-11	10.67
Tyrosine phosphorylation of STAT protein	GO:0007260	12	58	5.96E-11	10.23
Regulation of peptidyl-tyrosine phosphorylation	GO:0050730	17	186	9.62E-11	10.02

JAK-STAT cascade	GO:0007259	14	113	2.53E-10	9.60

Note: p-value represents the corrected p-value. Top ten canonical pathways are shown.

Table S5. Detailed functional annotation of pathway analysis.

**Pathway name**	**Database**	**ID**	**Input number**	**Background number**	**P-value**	**-Lg (P-value)**
Cytokine-cytokine receptor interaction	KEGG	hsa04060	34	265	1.19E-17	16.92
Interleukin signaling pathway	PANTHER	P00036	11	89	4.22E-09	8.37
Signaling by Interleukins	Reactome	REACT_22232	9	111	1.20E-06	5.92
Rheumatoid arthritis	KEGG	hsa05323	12	91	2.31E-06	5.64
Chemokine receptors bind chemokines	Reactome	REACT_15344	7	57	2.79E-06	5.55
Jak-STAT signaling pathway	KEGG	hsa04630	13	156	5.16E-05	4.29
AP-1 transcription factor network	PID	ap1_pathway	10	70	8.08E-05	4.09
Cytokine Signaling in Immune system	Reactome	REACT_75790	10	275	1.01E-04	4.00
Inflammatory bowel	KEGG	hsa05321	8	67	3.84E-0	3.42

disease					4	
Malaria	KEGG	hsa05144	7	49	4.40E-0<br>4	3.36

Note: p-value represents the corrected p-value. Top ten pathways are shown.

Supplemental Figures

Figure S1

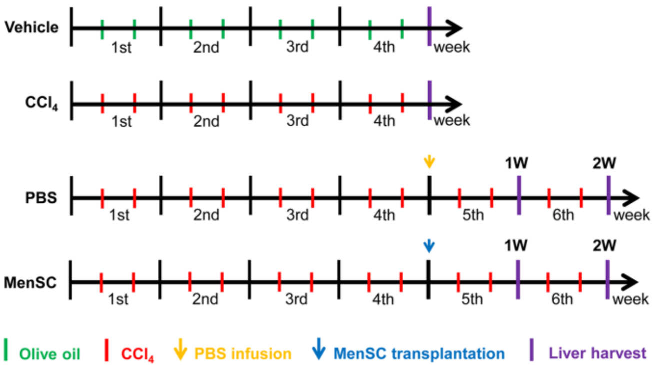

Figure S1. Experimental schematic of CCl_4-induced liver fibrosis in the mouse model. There were four groups: the vehicle group, CCl_4 group, MenSC group, and PBS group.

Figure S2

RayBio® Human Cytokine Antibody Array G6

	a	b	c	d	e	f	g	h	i	j	k	l	m	n
1	POS 1	POS 2	POS 3	NEG	NEG	Angiogenin	BDNF	BLC	BMP-4	BMP-6	CK b 8-1	CNTF	EGF	CCL11
2	POS 1	POS 2	POS 3	NEG	NEG	Angiogenin	BDNF	BLC	BMP-4	BMP-6	CK b 8-1	CNTF	EGF	CCL11
3	CCL24	CCL26	FGF-6	FGF-7	Flt-3 Ligand	CX3CL1	GCP-2	GDNF	CSF2	I-309	IFN-gamma	IGFBP-1	IGFBP-2	IGFBP-4
4	CCL24	CCL26	FGF-6	FGF-7	Flt-3 Ligand	CX3CL1	GCP-2	GDNF	CSF2	I-309	IFN-gamma	IGFBP-1	IGFBP-2	IGFBP-4
5	IGF-I	IL-10	IL-13	IL-15	IL-16	IL-1alpha	IL-1beta	IL-1ra	IL-2	IL-3	IL-4	IL-5	IL-6	IL-7
6	IGF-I	IL-10	IL-13	IL-15	IL-16	IL-1alpha	IL-1beta	IL-1ra	IL-2	IL-3	IL-4	IL-5	IL-6	IL-7
7	Leptin	LIGHT	MCP-1	MCP-2	MCP-3	MCP-4	M-CSF	MDC	MIG	MIP-1delta	MIP-3alpha	NAP-2	NT-3	PARC
8	Leptin	LIGHT	MCP-1	MCP-2	MCP-3	MCP-4	M-CSF	MDC	MIG	MIP-1delta	MIP-3alpha	NAP-2	NT-3	PARC
9	PDGF-BB	RANTES	SCF	SDF-1	TARC	TGF-beta1	TGF-beta 3	TNF-alpha	TNF-beta	NEG	NEG	NEG	NEG	NEG
10	PDGF-BB	RANTES	SCF	SDF-1	TARC	TGF-beta1	TGF-beta 3	TNF-alpha	TNF-beta	NEG	NEG	NEG	NEG	NEG

RayBio® Human Cytokine Antibody Array G7

	a	b	c	d	e	f	g	h	i	j	k	l	m	n
1	POS1	POS 2	POS 3	NEG	NEG	Acrp30	AgRP	Angiopoietin-2	Amphiregulin	Axl	bFGF	b-NGF	BTC	CCL-28
2	POS1	POS 2	POS 3	NEG	NEG	Acrp30	AgRP	Angiopoietin-2	Amphiregulin	Axl	bFGF	b-NGF	BTC	CCL-28
3	CTACK	Dtk	EGF-R	ENA-78	Fas/TNF RSF6	FGF-4	FGF-9	GCSF	GITR-Ligand	GITR	GRO	GRO-alpha	HCC-4	HGF
4	CTACK	Dtk	EGF-R	ENA-78	Fas/TNF RSF6	FGF-4	FGF-9	GCSF	GITR-Ligand	GITR	GRO	GRO-alpha	HCC-4	HGF
5	ICAM-1	ICAM-3	IGFBP-3	IGFBP-6	IGF-I SR	IL-1 R4/ST2	IL-1 RI	IL-11	IL-12 p40	IL-12 p70	IL-17	IL-2 Rapha	IL-6 R	IL-8
6	ICAM-1	ICAM-3	IGFBP-3	IGFBP-6	IGF-I SR	IL-1 R4/ST2	IL-1 RI	IL-11	IL-12 p40	IL-12 p70	IL-17	IL-2 Rapha	IL-6 R	IL-8
7	I-TAC	Lymphotactin	MIF	MIP-1alpha	MIP-1beta	MIP-3beta	MSP-alpha	NT-4	Osteoprotegerin	Oncostatin M	PIGF	sgp130	sTNF RII	sTNF-RI
8	I-TAC	Lymphotactin	MIF	MIP-1alpha	MIP-1beta	MIP-3beta	MSP-alpha	NT-4	Osteoprotegerin	Oncostatin M	PIGF	sgp130	sTNF RII	sTNF-RI
9	TECK	TIMP-1	TIMP-2	Thrombopoietin	TRAIL R3	TRAIL R4	uPAR	VEGF	VEGF-D	NEG	NEG	NEG	NEG	NEG
10	TECK	TIMP-1	TIMP-2	Thrombopoietin	TRAIL R3	TRAIL R4	uPAR	VEGF	VEGF-D	NEG	NEG	NEG	NEG	NEG

Figure S2. List of 120 cytokines (including human cytokine antibody array G6 and human cytokine antibody array G7) evaluated in the Human Cytokine G1000 array. POS, positive; NEG, negative.

Figure S3

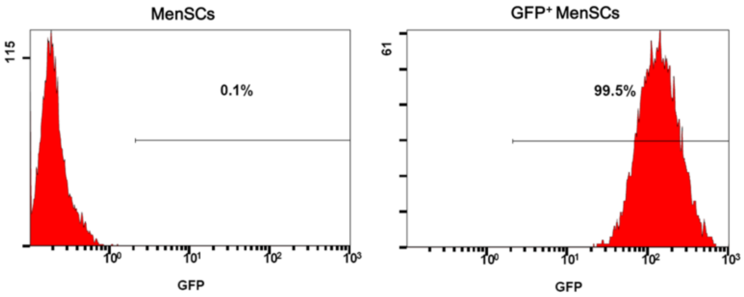

Figure S3. Confirmation of GFP expression in transduced MenSCs. The percentage of GFP-positive cells was measured by flow cytometry (n = 4).

Figure S4

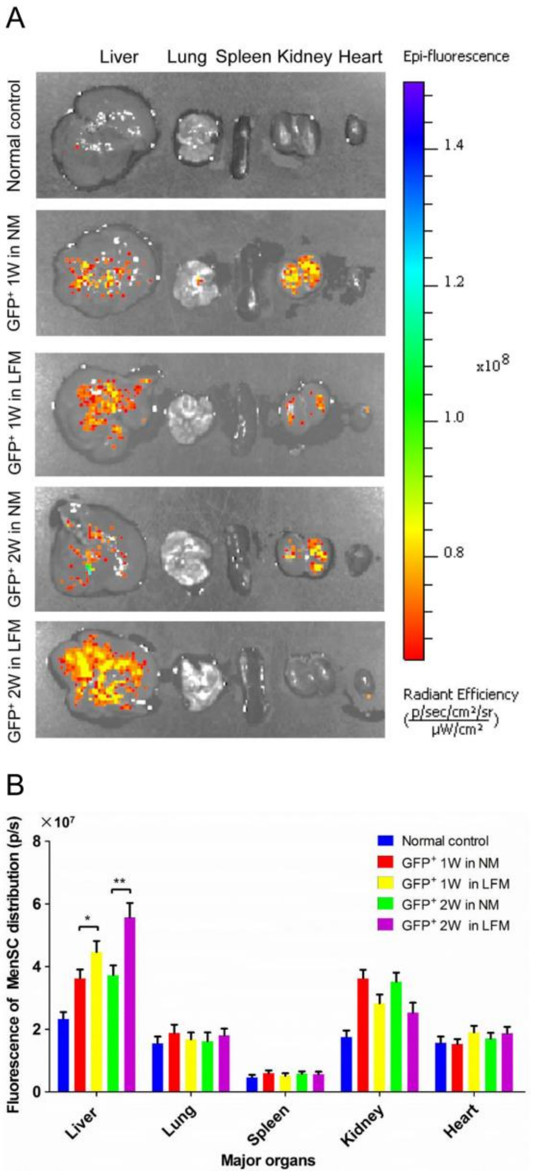

Figure S4. The distributions of MenSCs in the liver, lung, spleen, kidney, and heart with GFP^+ cell transplantation in normal mice (NM) and liver fibrosis mice (LFM). (**A**) Representative images are shown for normal control mice (without GFP), GFP^+ 1W and GFP^+ 2W in NM, and GFP^+ 1W and GFP^+ 2W in LFM. A total of 5×10^5 GFP^+ MenSCs in 1 mL PBS was injected into the tail vein of NM and LFM. (**B**) The quantities of MenSCs that migrated in different tissues after transplantation in NM and LFM are shown. Data represent the means ± SDs (n = 6). $^*P < 0.05$, $^{**}P < 0.01$.

Figure S5

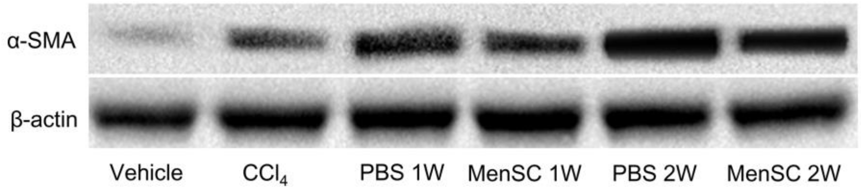

Figure S5. Detection of α-SMA expression by western blotting in different groups (vehicle, CCl_4, PBS 1W, MenSC 1W, PBS 2W, and MenSC 2W) in liver lysates. Representative images are shown. β-actin was used as an internal control.

Figure S6

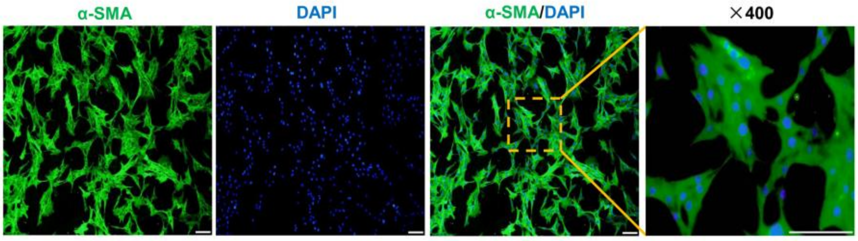

Figure S6. α-SMA immunostaining carried out to confirm the activation of LX-2 cells. More than 95% α-SMA expression represents the activated state of LX-2 cells. α-SMA (green) and DAPI (blue) are shown. Scale bar = 100 µm.

Figure S7

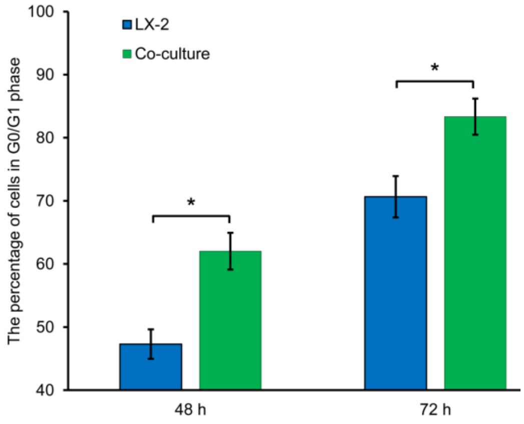

Figure S7. Percentages of LX-2 cells in the G0/G1 phase examined in single cultures (LX-2 group) and co-cultures (co-culture group) at 48 and 72 h. Data represent the means ± SDs (n = 4). *$P < 0.05$.

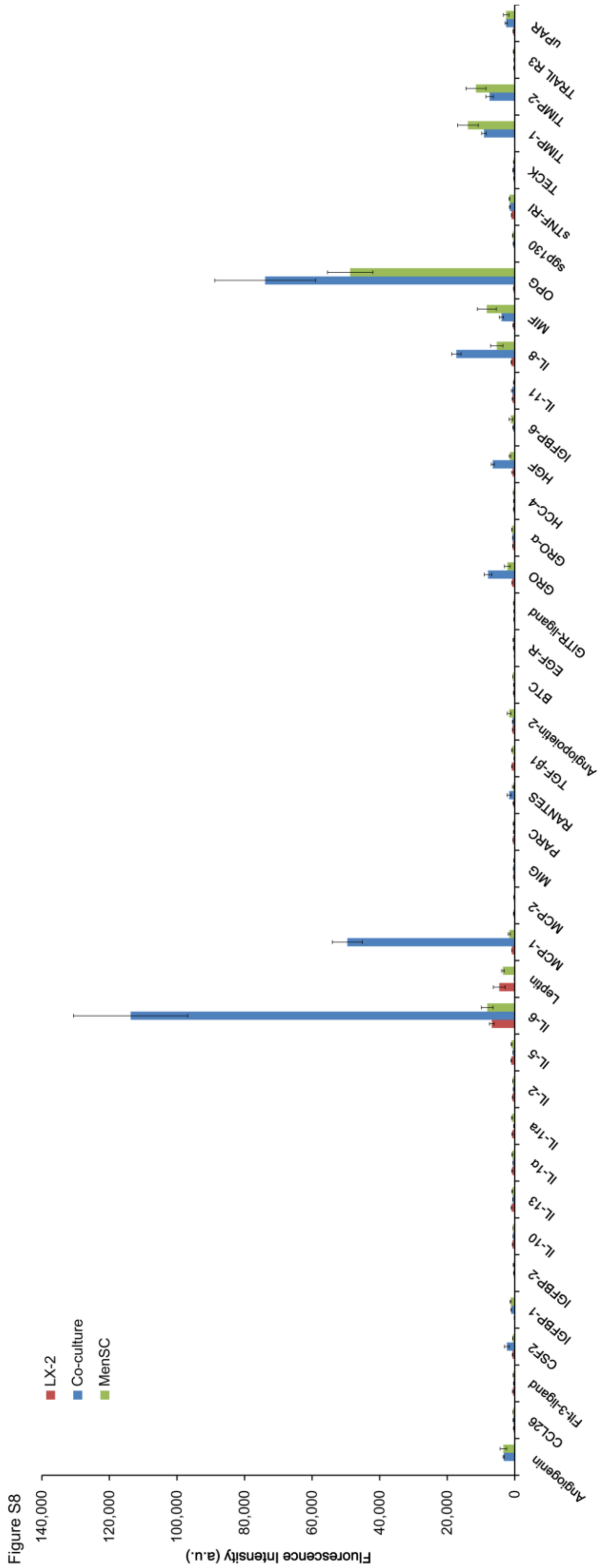

Figure S8. Normalized fluorescence intensities of differentially expressed cytokines shown for the LX-2 cells, MenSCs, and co-culture group. The values in the bar graph represent the means ± SDs (n = 4).
